# L’effet de l’extubation precoce apres chirurgie cardiaque pour la rehabilitation post opératoire

**DOI:** 10.11604/pamj.2017.28.81.11432

**Published:** 2017-09-27

**Authors:** Hichem Cheikhrouhou, Amine Kharrat, Rahma Derbel, Yesmine Ellouze, Karim Jmal, Hela Ben Jmaa, Mohamed Ali Elkamel, Imed Frikha, Abdelhamid Karoui

**Affiliations:** 1 Service d’Anesthésie Réanimation, CHU Habib Bourguiba, Sfax, Tunisie; 2 Service de Chirurgie Cardiovasculaire et Thoracique, CHU Habib Bourguiba, Sfax, Tunisie

**Keywords:** Extubation précoce, réhabilitation, chirurgie cardiaque, adulte, Early extubation, rehabilitation, cardiac surgery, adult

## Abstract

**Introduction:**

La réhabilitation post opératoire en chirurgie cardiaque est basée sur une prise en charge médico-chirurgicale visant à diminuer la durée de séjour hospitalier et les coûts de soins de cette chirurgie à haut risque. L’extubation trachéale précoce, définie par une extubation avant la sixième heure, constitue la pierre angulaire du Fast-tracking. L’objectif de notre étude est d’évaluer dans notre institution la pratique du fast-track et notamment de l’extubation trachéale précoce en chirurgie cardiaque adulte programmée.

**Méthodes:**

Nous avons conduit une étude descriptive incluant tous les patients âgés de plus 18 ans opérés consécutivement d’une chirurgie cardiaque programmée et qui ont été pris en charge en post-opératoire dans l’unité de réanimation post-opératoire du Service de chirurgie thoracique et cardiovasculaire du CHU HABIB BOURGUIBA de SFAX. Les critères d’inclusion des malades: âgés de 18 ans et plus, opérés d’une chirurgie cardiaque programmée, dont la prise en charge post-opératoire s’est faite dans l’unité de réanimation post-opératoire du service de chirurgie cardio-vasculaire et thoracique. Notre protocole d’anesthésie a été standardisé pour tous les patients: Propofol, Rémifentanil, Cisatracrium. Nous avons noté le délai d’extubation en post opératoire et les facteurs qui allongent ce délai d’extubation.

**Résultats:**

Nous avons colligé 200 observations de malades consécutifs qui ont bénéficié d’une chirurgie cardiaque programmée. Parmi ces malades 115 étaient opérés pour pontage aorto-coronaire, 79 pour chirurgie valvulaire et 6 pour chirurgie combinée ou autre. Les caractéristiques démographiques sont comparables. En post-opératoire, nous avons pu extuber 152 soit 76% de nos malades avant la sixième heure. Quarante-huit patients n’ont pas pu être extubés avant la sixième heure. Les principales causes de l’échec de l’extubation précoce sont : fortes doses de catécholamines, saignement, arythmie et les troubles neurologiques.

**Conclusion:**

Nous avons pu démontrer que la réalisation de la réhabilitation post opératoire était possible dans notre institution et tous les patients bénéficiant d’une chirurgie cardiaque réglée devraient être candidats à l’extubation précoce.

## Abstract (en)

**Introduction:**

Postoperative rehabilitation after cardiac surgery is based on medical-surgical management in order to reduce the lenght of stay in hospital and the costs of this high risk surgery. Early tracheal extubation (within the first 6 hours) is the cornerstone of fast-track surgery. Our study aimed to evaluate fast-track practice and early tracheal extubation in scheduled cardiac surgery for adult patients in our Institution.

**Methods:**

We conducted a descriptive study including all patients aged over 18 years who consecutively had undergone scheduled cardiac surgery and postoperative treatment in the post-operative intensive care unit in the Department of Thoracic and Cardiovascular Surgery at the Habib Bourguiba University Hospital, Sfax. Inclusion criteria were: patients aged 18 years and older who had undergone scheduled cardiac surgery and postoperative treatment in the post-operative intensive care unit in the Department of Thoracic and Cardiovascular Surgery. Standardized anaesthetic protocol was used in all cases: propofol, remifentanil, cisatracrium. We recorded the mean postoperative extubation time and the factors affecting extubation time.

**Results:**

We collected data from 200 patients who consecutively had undergone scheduled cardiac surgery. Among these patients, 115 underwent coronary artery bypass surgery, 79 valvular surgery and 6 combined surgery or another surgical procedure. Patients’ demographic characteristics were comparable. 152 patients (76%) underwent postoperative extubation within the first 6 hours. 48 patients couldn’t be extubated within the FIrst 6 hours. The main causes of early extubation failure were: catecholamines in high doses, bleeding, arrhythmia and neurological disorders.

**Conclusion:**

Our study demonstrates that postoperative rehabilitation can be performed in our Institution and that all patients undergoing scheduled cardiac surgery should be candidates for early extubation.

## Introduction

L’anesthésie en chirurgie cardiaque, se basant sur une évaluation préopératoire rigoureuse et disposant d’un arsenal de techniques et de moyens de monitorage modernes ainsi que de nouvelles molécules à cinétique rapide a pour objectif de maintenir une stabilité hémodynamique peropératoire garante d’une réduction de la morbidité post-opératoire et d’envisager une extubation trachéale précoce dans le cadre de la réhabilitation post opératoire. L’extubation trachéale précoce constitue la pierre angulaire des parcours rapides en chirurgie cardiaque programmée, elle est définie par une extubation avant la sixième heure post-opératoire [1]. Parallèlement, le développement de techniques chirurgicales mini -invasives contournant le recours à la circulation extracorporelle a permis chez des patients sélectionnés de diminuer les complications post-opératoires et la durée de séjour en réanimation. Enfin, la réanimation post-opératoire basée sur la prévention et la détection des complications et une stratégie efficace et précoce de l’analgésie aboutissant à l’extubation précoce constitue le dernier maillon d’une chaîne de soins dont l’équilibre est précaire et où toute défaillance pourrait amener l’équipe de soins à renoncer à la stratégie de Fast-tracking. Si la pratique du Fast-tracking en Amérique du nord et en Europe remonte à plusieurs années [2], son application dans d’autres pays [3] et particulièrement en Tunisie ne date que de peu de temps. Nous nous proposons dans ce travail d’analyser les pratiques relatives aux parcours rapides en chirurgie cardiaque adulte programmée au CHU. Habib Bourguiba de Sfax. L’objectif principal de notre étude est d’évaluer dans notre institution la pratique du fast track et notamment de l’extubation trachéale précoce en chirurgie cardiaque adulte programmée.

## Méthodes

Nous avons conduit une étude descriptive incluant tous les patients âgés de plus 18 ans opérés consécutivement d’une chirurgie cardiaque programmée et qui ont été pris en charge en post-opératoire dans l’unité de réanimation post-opératoire. Nous avons inclus dans notre étude les patients: âgés de 18 ans et plus, opérés d’une chirurgie cardiaque programmée dont l’indication était: une pathologie coronaire, une pathologie valvulaire ou autre (aorte thoracique, kyste ou tumeur du cœur). Nous avons recueilli les paramètres démographiques des patients, les antécédents médicaux et chirurgicaux et les traitements en cours. L’évaluation préopératoire était basée sur: les données de l’examen clinique (présence de dyspnée, angor, stade NYHA et classe ASA), les examens biologiques (groupage sanguin, RAI, numération sanguine, bilan d’hémostase, fonction rénale), un électrocardiogramme, la radiographie thoracique, une échographie cardiaque, un doppler des vaisseaux du cou, les données de la coronarographie et l’évaluation du risque chirurgicale par l’Euroscore.

En peropératoire, nous avons noté: la durée et la nature de l’intervention (chirurgie valvulaire, chirurgie de pontage aorto coronaire). Un protocole d’anesthésie était préétabli pour la chirurgie cardiaque programmée. La prémédication est faite par l’hydroxizine la veille de l’acte et 2 heures avant le bloc. Le monitorage: électrocardioscope à 5 électrodes, Saturation pulsée en O2, température, diurèse, pression veineuse centrale, P et CO2, monitorage des concentrations inhalées des halogénés, glycémie pour les diabétiques. L’induction par hypnomidate (0,3 à 0,5 mg/kg), remifentanil 1μg/kg et cis-atracurium 0,15 mg/kg. L’antibioprophylaxie par le cefazoline. L’entretien de l’anesthésie: propofol et remifentanil au PSE et réinjection de curare toutes les 30 mn. A la fin de l’intervention: Paracétamol 1 gr et sulfate de morphine 0,15 mg/kg 30 min avant l’arrêt de la perfusion du rémifentanil. Pour la conduite de la circulation extracorporelle (CEC) et protection myocardique, nous avons prélevé la durée de la CEC, la durée du clampage aortique et la durée de l’assistance circulatoire ainsi que la nature de la solution de cardioplégie (cristalloïde ou sanguine) et la voie de son administration (antérograde ou rétrograde), le départ et l’arrêt de la CEC sont décidés en collaboration avec l’équipe chirurgicale. Nous avons noté en peropératoire la a survenue d’une instabilité hémodynamique, le type et la dose de catécholamine utilisée, la survenue d’un saignement important, transfusion peropératoire, des troubles du rythme, de la conduction et de troubles de la repolarisation.

En post opératoire, et après l’obtention des résultats du bilan postopératoire immédiat, l’extubation trachéale est envisagée chez les patients lorsque des critères prédéfinis étaient réunis (Tableau 1). Nous avons noté le délai d’extubation par rapport à l’admission en réanimation. Nous avons recueilli les complications post opératoires: le saignement, une transfusion, les complications cardiovasculaires (HTA, hypotension, hypo débit, ischémie myocardique, infarctus du myocarde, tamponnade, AC/FA, troubles de la conduction, OAP cardiogénique), les complications respiratoires (atélectasies, pneumopathie, réintubation, épanchement pleural), les complications neurologiques (retard de réveil, agitation, AVC, convulsions), les complications rénales (IRA, dialyse) et la nécessité de reprise chirurgicale. En l’absence de complications postopératoires et devant une stabilité hémodynamique, le patient était mis au fauteuil et sa déambulation précoce est entamée. La sortie de la réanimation était possible si des critères de sortie de la réanimation étaient réunis (Tableau 2). Nous avons noté la durée du séjour en réanimation.

**Tableau 1:** Conditions requises pour l’extubation trachéale précoce

Système nerveux central: patient réveillé, coopérant, avec réflexe de toux
Système cardiovasculaire: stabilité hémodynamique sans arythmie
Système respiratoire: sevrage du respirateur en mode VS-AI-PEP puis épreuve sur pièce enT, capacité vitale > 6 ml/kg, PaO2>80 mmHg avec FiO2<0,5, pH>7,35
Température > 36,5°C sans frisson
Pertes sanguines par les drains médiastinaux <100 ml/h depuis >2 heures
Circulation périphérique et diurèse satisfaisante > 1 ml/kg/h

**Tableau 2:** Conditions requises pour la sortie de réanimation

Cœur	Absence d’ischémie et d’infarctus myocardique majeur; Rythme sinusal, arythmie traitée ou contrôlé; Absence de signes de bas débit, de surcharge volémique; arrêt des agents vasopresseurs et inotropes.
Poumon	SaO2> ou= 94% sous O2<6l/mn; absence de condensation pulmonaires et d’épanchements
Cerveau	Patient orienté, coopérant
Rein	Diurèse>à 0,5ml/kg/h, créatinémie<150% des valeurs préopératoires
*Autres*	Absence de douleur, absence de signes infectieux.

## Résultats

Nous avons colligé 200 patients dont 115 étaient opérés pour pontage aorto coronaire, 79 pour chirurgie valvulaire et 6 pour chirurgie combinée ou autre. La population coronaire était plus âgée (63,1±9,8 ans contre 42,5±13,9 pour la population valvulaire) avec plus d’antécédents d’HTA, de BPCO, d’AVC et de diabète. L’arythmie complète par fibrillation auriculaire était plus fréquente chez la population valvulaire. Un Euroscore supérieur à 6 était retrouvé chez 46% de nos patients. L’Euroscore moyen était plus élevé dans la population coronaire (Tableau 3).

**Tableau 3:** Caractéristiques globales de la population

	Population Totale	Chirurgie coronaire	Chirurgie valvulaire	Chirurgie combinée et autre
Patients (n)	200	115	79	6
Age moyen ± écart type (ans)	54,7±15,8	63,14 ± 9,8^+^	42,52±13,9	48,6 ±6,3
Âge >70ans (%)	19	25,2	11,4	0
Sexe masculin/féminin	131/69	82/33	44/35	1/6
HTA^1^ (%)	48,5	76,52	10,12	16,6
Diabète (%)	38	62,6	5,06	0
ACFA^2^ (%)	16,5	3,47	34,17	33,3
IRC^3^ (%)	8,5	7,82	7,6	16,6
BPCO^4^ (%)	12,5	18,26	3,8	16,2
AVC^5^ (%)	8	10,4	5,06	0
FEVG^6^ < 50% (%)	36	40,8	29,11	33,3
EUROSCORE (points)	5,51±3,32	6,55±3,2^+^	4,15±2,9	4,76±2,1
EUROSCORE > 6(%)	46	46,08	45,56	33,33
ASA^7^>III (%)	57	57,39	58,22	33,33
NYHA^8^>III (%)	22	22,6	21,51	16,66

1 hypertension artérielle;

2 arythmie complète par fibrillation atriale;

3 insuffisance rénale chronique;

4 bronchopneumopathie chronique obstructive;

5 accident vasculaire cérébral;

6 fraction d’éjection du ventricule gauche;

7 American Society of Anesthesiologists;

8 New York Heart Association.

+ : p<0,05

Pour la chirurgie des coronaires, l’âge moyen, la créatinémie et l’Euroscore étaient plus élevés chez les patients opérés à cœur battant. En revanche, la fraction d’éjection du ventricule gauche, le nombre moyen de pontages et de culots globulaires transfusés étaient plus élevés dans la population opérée avec CEC (Tableau 4). Les durées de CEC et de clampage étaient légèrement plus longues en chirurgie coronaire (Tableau 5). L’utilisation d’antifibrinolytique était plus fréquente en chirurgie coronaire avec un recours plus fréquent à l’acide tranexamique. La sortie de la CEC était plus fréquemment difficile et sous fortes doses de catécholamines. La durée de la chirurgie était significativement plus basse pour la chirurgie coronaire à cœur battant (Tableau 6).

**Tableau 4:** Différences associées à l’utilisation ou non d’une CEC en chirurgie coronaire

	SANS CEC	AVEC CEC	P
*Effectif (n)*	30	85	
*Âge moyen (ans)*	62,38±9,7	64,13 ± 9,8	0,07
*Créatinémie (μmol/l)*	119,6 ± 47,5	98,3 ± 33,2	0,02
*Fraction d’éjection du ventricule gauche (%)*	52,6±12,3	56,4±13,1	0,1
*Nombre de pont /patient(n)*	1,99±0,54	2,87±0,59	0,02
*Transfusion (CG/ patient)*	0,68±0,54	3,56±1,06	0,001
*EUROSCORE (points)*	6,68±3,21	6,34±3,2	0,06

**Tableau 5:** Réalisation de la CEC selon le geste

	Chirurgie coronaire	Chirurgie valvulaire
Durée CEC (min)	95,6 ± 23	84,8 ± 23^+^
Durée clampage (min)	77,6± 20,3	74,6 ± 14,9
Ultrafiltration(n)	1	2
Acide tranexamique(n)	66	34^+^
Aprotinine(n)	7	9
Sortie CEC sous forte dose de catécholamines (n)	11	23^+^

+ p<0,05

**Tableau 6:** Durée de la chirurgie selon le geste

	RVAO	RVM	DRV	PACCEC	PACCB
Temps de chirurgie (min)	209,7±44	212,4±35,5	247,9±55,3	264,9±45,5	171,2±28,6^+^

+ p<0,05

En post opératoire, nous avons pu extuber 76 % de nos malades avant la sixième heure avec un taux significativement plus élevé en chirurgie coronaire à cœur battant (PACCB) et chirurgie valvulaire de remplacement de la valve aortique (RVAo). La durée moyenne de ventilation mécanique (DMVM) était de 8,2±8,5 heures avec une durée plus courte pour les malades opérés par PACCB, la durée moyenne de séjour en réanimation était de 3,6j ± 4,06 avec un pourcentage de malades ayant séjourné plus que 48h de 42,5 %. De même la durée de séjour et le pourcentage de malades dont le séjour s’est prolongé au-delà de 48 h étaient plus bas dans la population opérée pour PACCB et pour RVAo.

La comparaison du groupe des patients extubés précocement à ceux dont l’extubation précoce a échoué ne montre pas de différences entre les deux groupes en termes d’âge, de sexe et de la moyenne de l’Euroscore. Par contre la durée de la chirurgie et la durée de la CEC étaient plus prolongées dans le groupe échec de l’extubation précoce. De même le saignement ainsi que la fréquence de transfusion et le nombre de culots globulaires transfusés étaient plus élevés dans le même groupe. Le recours à une réintubation pour détresse respiratoire aigüe n’était pas plus fréquent dans le groupe extubation précoce. En revanche on a noté moins de complications pulmonaires infectieuses dans le même groupe. La durée moyenne du séjour en réanimation était plus courte dans le groupe extubation précoce (Tableau 7). Les principales causes de l’échec de l’extubation précoce étaient la sortie de la CEC sous forte dose de catécholamines chez 34 malades, un saignement important chez 6 malades, une arythmie chez 4 malades , la mise en place d’une CPIA chez 2 malades et les troubles neurologiques chez 2 malades.

**Tableau 7:** Délai d’extubation et durée du séjour en réanimation en fonction du type de chirurgie

	Chirurgie coronaire		Chirurgie valvulaire		
	PACCEC^1^ (n=85)	PACCB^2^ (n=30)	RVAo^3^ (n=23)	RVM^4^ (n=33)	DRV^5^ (n=23)
DMVM^6^ (heures)	9,2±10,6	3,3±2^+^	5,6±4,7^+^	12±25,8	10,±20,2
Ext pr^7^ (n (%))	60 (70,6%)	29 (96,6)^+^	20(87%)^+^	25(83,3%)	18(78,2%)
Séjour en réanimation (jours)	4±4,6	2,6±1,8^+^	2,7±1,08^+^	4,8±6,53	3,8±1,81
Séjours > 48 h (n(%))	45 (52,9%)	4 (13,3%)^+^	7(30,4%)^+^	14(42,4%)	15(65,2%)

+ p<0,05;

1 pontage aortocoronarien avec cec;

2 pontage aortocoronarien à cœur battant;

3 remplacement valve aortique;

4 remplacement valve mitrale;

5 double remplacement valvulaire;

6 durée de ventilation mécanique;

7 extubation précoce

## Discussion

La réhabilitation postopératoire est un concept dont Kehlet a proposé une définition: c’est une approche multidisciplinaire de la période postopératoire visant au rétablissement rapide des capacités physiques et psychiques antérieures d’un patient opéré [4]. Ce concept a été développé initialement dans la chirurgie digestive, particulièrement dans la chirurgie colorectale [5]. Son application pour la chirurgie cardiaque s’avère très utile devant une chirurgie de longue durée, avec recours ou non à la circulation extracorporelle. Parmi les principales mesures de la réhabilitation post chirurgie cardiaque l’extubation précoce qui est favorisée par des facteurs per opératoires (stabilité hémodynamique, saignement minime, courte durée de CEC, faibles doses de catécholamines) et post opératoires (analgésie post opératoire, bon état neurologique, stabilité hémodynamique, faibles doses de catécholamines) [6]. Cette approche dite « Fast Track » s’est développée en réponse à l’amélioration des techniques chirurgicales. Celle-ci est apparue depuis une dizaine d’années en vue d’une réduction des coûts hospitaliers [7].

La réussite de ce concept dépend des antécédents du patient proposé pour la chirurgie cardiaque pour prédire le résultat de cette chirurgie. L’âge, l’insuffisance rénale, le sexe féminin, la durée de chirurgie et la durée du clampage vasculaire ont été associés avec un échec de réhabilitation post opératoire [8]. L’utilisation de score de gravité en chirurgie cardiaque a permis la stratification des patients et d’évaluer leurs risques éventuels pour une meilleure prise en charge. Parmi ces scores nous avons utilisé l’Euroscore qui a été publié en 1999 d’après une étude de Nashef [9]. Ce score permet de prédire le succès de réhabilitation post opératoire, l’augmentation de ce score d’associe avec une diminution de probabilité d’achever le processus de réhabilitation correctement [10]. L’extubation trachéale précoce est définie comme l’extubation dans une fenêtre de temps de la deuxième à la dixième heure postopératoire. Depuis plus de 20 ans des publications de plus en plus nombreuses ont souligné l’intérêt d’une extubation précoce en termes de diminution des complications respiratoires postopératoires. Johnson [11] comparait 2 groupes de patients opérés de pontages coronariens ; le groupe extubé précocement avait moins d’atélectasie et une meilleure fonction respiratoire que le groupe extubé tardivement. Sur une cohorte d’hommes âgés de plus de 60 ans le protocole d’extubation précoce permettait une réduction du taux de pneumopathie nosocomiale (7,3% versus 14,7%) [12]. L’extubation améliore également la performance ventriculaire en diminuant la pression intra-pleurale et en augmentant le diamètre du ventricule gauche en fin de diastole ce qui conduit à une augmentation du débit cardiaque [13]. Selon Reis et coll. il n’y avait pas plus de complications en postopératoire chez les patients anesthésiés avec la méthode « Fast Track » que ceux ayant bénéficié d’une anesthésie standard [14]. Quant à Cheng il démontrait dans une étude prospective que l’extubation précoce permettait de réduire la durée d’hospitalisation sans augmenter les complications postopératoires [15].

## Conclusion

La réhabilitation post opératoire est un concept très intéressant pour la chirurgie cardiaque. Sa réussite nécessite une collaboration multidisciplinaire. Dans ce cadre, l’extubation précoce s’avère très utile pour réussir ce concept sous réserve de limiter les complications per et post opératoire. Mais la réussite de la réhabilitation nécessite encore des protocoles plus avancés.

### Etat des connaissances actuelles sur le sujet

- L’extubation post chirurgie cardiaque est retardée dans quelques centres de chirurgie;
- L’extubation retardée aide à régler les troubles biologiques et respiratoires.

### Contribution de notre étude à la connaissance

- Les effets bénéfiques de l’extubation précoce;
- Les facteurs limitants l’extubation précoce.

## Conflits d’intérêts

Les auteurs ne déclarent aucun conflit d'interêts.
